# Perspective on quantitative phase imaging to improve precision cancer medicine

**DOI:** 10.1117/1.JBO.29.S2.S22705

**Published:** 2024-03-26

**Authors:** Yang Liu, Shikhar Uttam

**Affiliations:** a University of Illinois Urbana-Champaign, Beckman Institute for Advanced Science and Technology, Cancer Center at Illinois, Department of Bioengineering, Department of Electrical and Computer Engineering, Urbana, Illinois, United States; b University of Pittsburgh, Departments of Medicine and Bioengineering, Pittsburgh, Pennsylvania, United States; c University of Pittsburgh, Department of Computational and Systems Biology, Pittsburgh, Pennsylvania, United States

**Keywords:** quantitative phase imaging, interferometry, computational imaging, cancer research

## Abstract

**Significance:**

Quantitative phase imaging (QPI) offers a label-free approach to non-invasively characterize cellular processes by exploiting their refractive index based intrinsic contrast. QPI captures this contrast by translating refractive index associated phase shifts into intensity-based quantifiable data with nanoscale sensitivity. It holds significant potential for advancing precision cancer medicine by providing quantitative characterization of the biophysical properties of cells and tissue in their natural states.

**Aim:**

This perspective aims to discuss the potential of QPI to increase our understanding of cancer development and its response to therapeutics. It also explores new developments in QPI methods towards advancing personalized cancer therapy and early detection.

**Approach:**

We begin by detailing the technical advancements of QPI, examining its implementations across transmission and reflection geometries and phase retrieval methods, both interferometric and non-interferometric. The focus then shifts to QPI’s applications in cancer research, including dynamic cell mass imaging for drug response assessment, cancer risk stratification, and *in-vivo* tissue imaging.

**Results:**

QPI has emerged as a crucial tool in precision cancer medicine, offering insights into tumor biology and treatment efficacy. Its sensitivity to detecting nanoscale changes holds promise for enhancing cancer diagnostics, risk assessment, and prognostication. The future of QPI is envisioned in its integration with artificial intelligence, morpho-dynamics, and spatial biology, broadening its impact in cancer research.

**Conclusions:**

QPI presents significant potential in advancing precision cancer medicine and redefining our approach to cancer diagnosis, monitoring, and treatment. Future directions include harnessing high-throughput dynamic imaging, 3D QPI for realistic tumor models, and combining artificial intelligence with multi-omics data to extend QPI’s capabilities. As a result, QPI stands at the forefront of cancer research and clinical application in cancer care.

## 1 Introduction

Quantitative phase imaging (QPI) represents an important advancement in the field of microscopy, offering a label-free technique that translates phase differences, or optical path length variations introduced by a sample, into detectable intensity contrasts. This unique capability reveals the intrinsic contrast based on the refractive indices of the sample components. The origins of phase contrast microscopy can be traced back to the pioneering work of Frits Zernike, awarded the Nobel Prize in Physics in 1953,^1^ which heralded a new era in biological research by allowing the visualization of transparent biological specimens without staining. This innovation has been instrumental in enabling biologists to examine live cells in their natural, undisturbed states.

Although traditional phase microscopy opened new avenues for visualizing live cells, it was initially confined to qualitative observations, lacking the sufficient precision for detailed quantitative analyses of biological processes. The transition from qualitative to quantitative phase imaging, pioneered by Popescu and others,^2^^–^^5^ marked a significant shift in this field. QPI not only facilitates visualization but also enables accurate quantification of optical path differences. Over the past two decades, this field has seen an explosion in the development of various techniques aimed at extracting quantitative phase information. These methodologies encompass both interferometric and non-interferometric strategies and are employed in diverse configurations including transmission and reflection modes. These techniques have a wide range of applications, extending from the study of thin, cultured cell monolayers and tissue sections to the imaging of thick, three-dimensional embryos and whole organisms. They offer high-speed, high-throughput, and high-resolution capabilities, serving diverse areas within both basic and translational biomedical research.

Building upon these diverse applications, the potential of QPI extends significantly into precision cancer medicine. This field synergizes the tailored approach of precision oncology—where treatments are based on the genetic and molecular profiles of individual tumors—with the proactive strategies of precision prevention, which aim to identify and mitigate risk factors and detect cancer at its earliest stages in individual patients. In these critical domains, QPI’s ability to non-invasively assess the biophysical properties of cancer cells in real time could be important in identifying early indications of drug resistance or metastatic potential. The application of QPI in monitoring cell mass dynamics provides insight into tumor growth and treatment responses at the cellular level, which is essential for the development of personalized treatment strategies. Moreover, the precision of QPI could enhance cancer surveillance programs by detecting subtle changes in cellular behaviors that are indicative of treatment efficacy or early signs of relapse. In the field of cancer prevention, QPI could play a significant role in identifying early cellular alterations that precede malignancy, thereby contributing to prevention strategies for individuals at high risk.

This perspective will outline the technical advancements and applications of QPI in the context of cellular and tissue imaging, with a special emphasis on cancer research. We will discuss existing challenges and explore strategies for the incorporation of QPI to significantly enhance cancer precision medicine.

## 2 Technical Implementations of QPI

### 2.1 Configuration and Image Contrast of QPI Systems

QPI encompasses various technical implementations aimed at extracting quantitative phase information from intensity images. The central mechanism in all phase imaging techniques involves converting phase variations in the sample into detectable intensity variations at the camera sensor. This section highlights key technical approaches in QPI, paving the way for our discussion on future perspectives. A more exhaustive review can be found in the literature.^2^

**Fig. 1:**
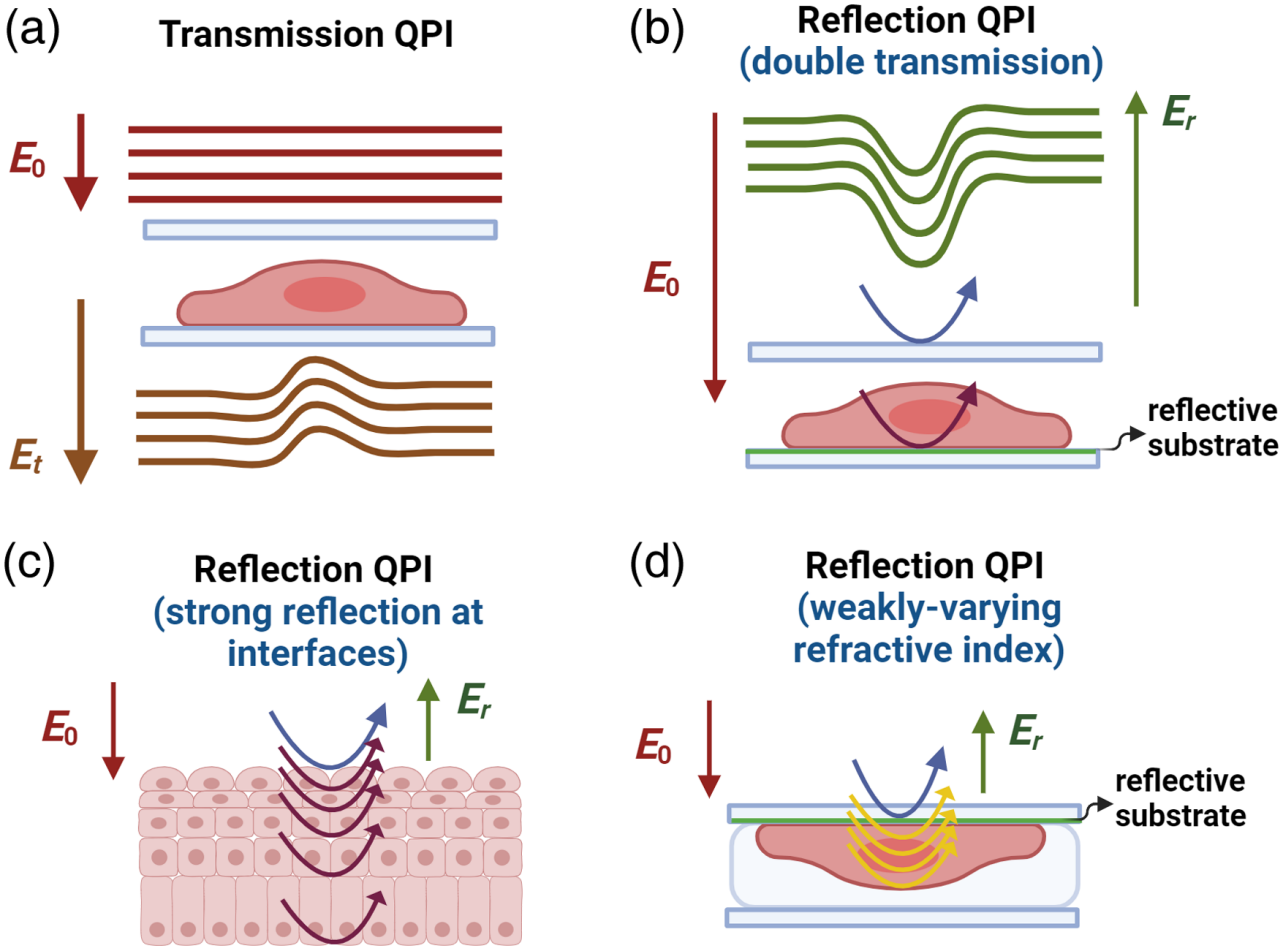
Example of light path in (a) transmission phase imaging and (b-d) three different scenarios in reflection phase imaging configurations, including (b) double-transmission case with a strong reflective substrate, (c) a case of strong multi-layer reflections at interfaces with a large refractive index mismatch, and (d) a case of weakly-varying refractive indices within the sample where the reflection from the reflective surface serves as reference wave interferes with the weak backscattered light from heterogenous refractive index changes within the sample.

QPI systems generally adopt one of two configurations. The first, transmission QPI, utilizes a transmission mode where illumination light passes through the sample and the transmitted light is captured by the sensor [Fig. 1(a)]. The second, reflection QPI, operates in a reflection configuration [Figs. 1(b)–1(d)], where part of the illumination light reflects off surfaces with strong refractive index mismatches, and the rest, transmitted into the sample, scatters due to internal refractive index variations within the sample. In both types, the wave at the detector is a superposition of a reference wave (light in the absence of the sample) and the sample wave. Systems typically use either monochromatic or broadband light sources. Partially coherent light with nearly collimated illumination enhances interference contrast. The detected intensity of superposed waves in either transmission or reflection QPI can be expressed as: $I(x,y)={\left|E_{r}(x,y)\right|}^{2}+{\left|E_{s}(x,y)\right|}^{2}+2R[E_{r}^{*}(x,y)E_{s}(x,y)]$.^6^ The first two terms denote the DC components from the reference and the sample intensity respectively, and the interference term carries the phase information.

Quantitatively, phase measures the delay of the wavefront as light travels through the sample. In transmission QPI, light traverses the entire sample, accumulating phase shifts relative to the reference wave. These shifts can be quantified and linked to the physical parameters of the sample via the expression: $\varphi (x,y)=\int \frac{2\pi }{\lambda }(n_{s}(x,y,z)-n_{r})dz$, where λ is the wavelength of light, $n_{s}$ and $n_{r}$ are the refractive indices of the sample and the homogeneous medium, respectively, and z is the axial position. The pixel-wise value of the quantitative phase image, often expressed as optical path length (OPL) map, is $\mathrm{OPL}(x,y)=(n_{s}(x,y)-\overset{¯}{n_{r}})d(x,y)$, where d(x,y) is the physical thickness at each location (x,y) for thin biological samples. This OPL, also referred to as phase shift, couples the physical thickness with the refractive index difference between the sample and the medium, potentially leading to ambiguity in interpreting cellular properties.

**Fig. 2:**
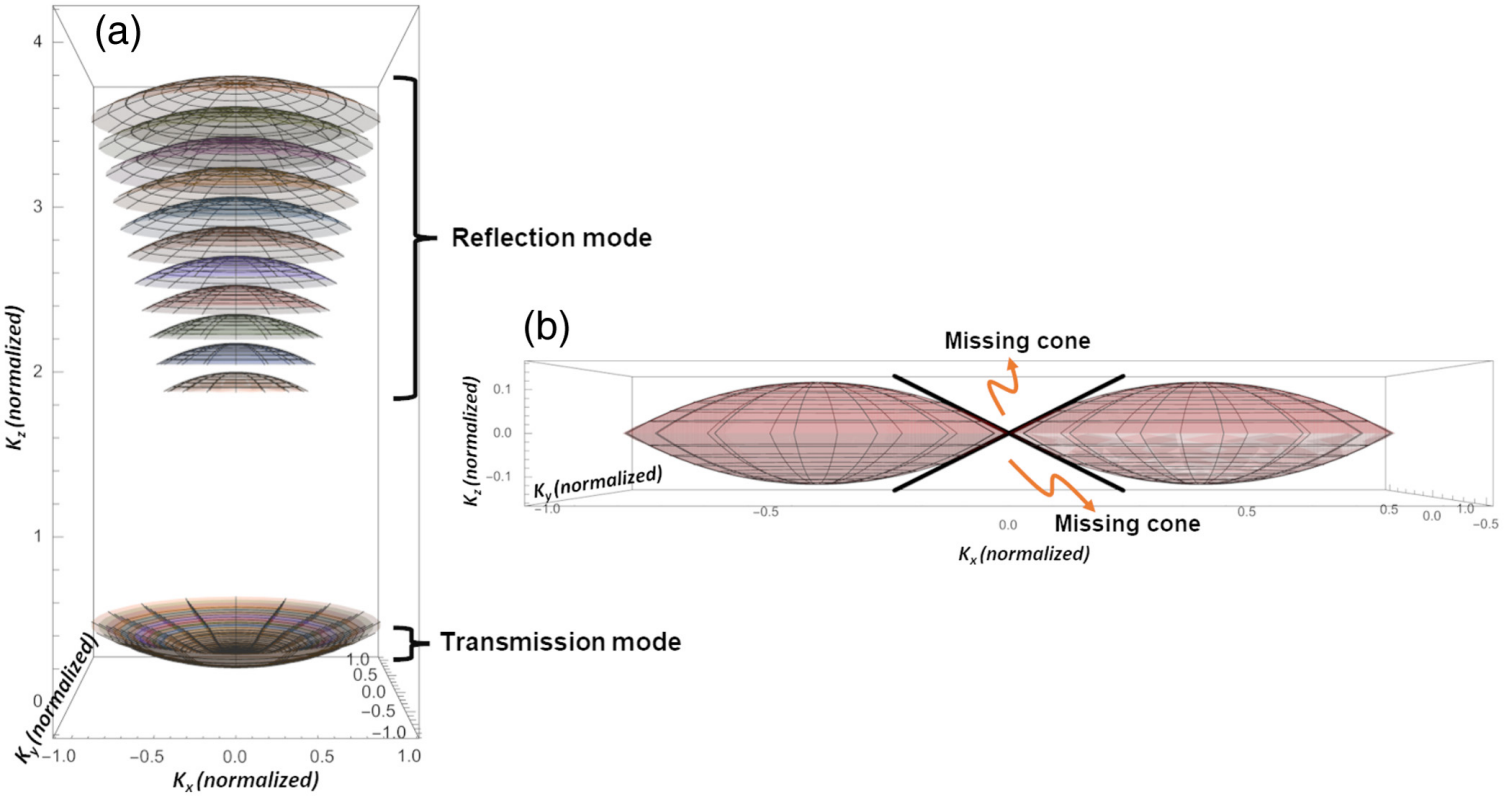
Frequency domain support of QPI modalities. (a) Transmission and reflection mode QPI. In the reflection mode, the spectral bandwidth of the light source provides axial ($K_{z}$) frequency support resulting in its ability to perform depth-resolved QPI. In contrast, this frequency support collapses to the origin in the transmission mode with $K_{z}=0$ for all source wavelengths. As a result, the transmission mode does not perform depth-resolved QPI but is able to capture the average quantitative phase with its frequency support centered around the origin. Reflection mode is unable to capture the latter due to a lack of frequency support at the origin. Instead, it captures higher frequency structures from the sample due to its frequency support away from the origin. Both provide similar, numerical aperture-dependent lateral resolution, as indicated by the lateral spread of the frequency support. (b) Optical diffraction tomography. Missing cone in ODT frequency support reduces axial resolution of ODT based 3D QPI imaging.

Reflection QPI differs significantly in principle from transmission QPI. Due to the near reversal of axial directions between illumination and backscattered wave vectors, reflection geometry covers higher spatial frequencies [or $k_{z}$ in K-space, or Fig. 2(a)]. However, reflection microscopy lacks access to low $k_{z}$ ranges captured in the transmission mode [Fig. 2(a)], resulting in its inability to capture the average refractive index detected in transmission geometry.^7^

In some cases, reflection QPI modalities, despite being in the physical configuration of reflection mode, are designed to mimic double transmission [Fig. 1(b)], offering image contrasts from cumulative phase shifts similar to transmission QPI. For example, when samples are on highly reflective substrates, the predominant signal is from the substrate reflection, with backscattered light within the sample being negligible. This results in a quantitative phase image that effectively doubles the OPL difference between the sample and surrounding medium, like double-pass transmission. The image contrast from biological samples resembles that of transmission QPI and may even provide slightly enhanced contrast due to the doubled OPL through the sample.^8^^–^^10^

**Fig. 3:**
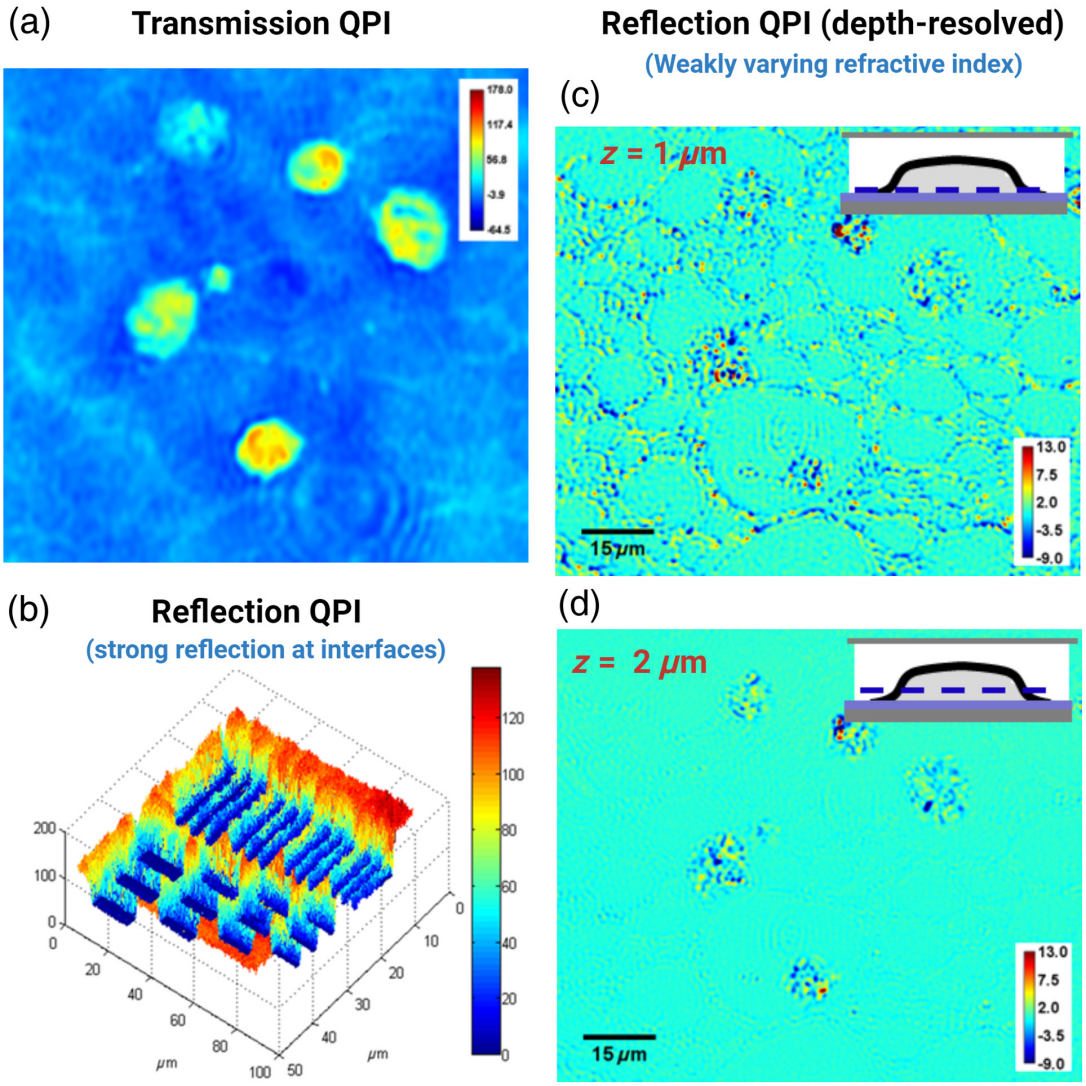
Representative images from (a) transmission QPI of cells embedded in resin-based substrates, (b) reflection QPI of a USAF target in the presence of strong interfaces, and (c) and (d) reflection QPI of weakly scattering objects (cells embedded in resin-based substrates) in the absence of strong interfaces at different depths.

In certain reflection QPI systems, strong backscattered light from internal interfaces enables extraction of quantitative phase information at these interfaces [Fig. 1(c)], providing depth selectivity. Broadband light sources used in this context resemble optical coherence tomography, where the inherent coherence gate offers depth selectivity. Depending on the location of coherence gate, the quantitative phase shift between the reference and sample wave can be measured either at the sample surface^11^ [see Fig. 3(b) as an example] or within subsurface tissue layers with strong refractive index mismatches.^12^

There are certain cases where the sample lacks strong reflective interfaces, and after surface reflections have been redirected, the reflection QPI captures predominant signals from backscattered light of weakly varying refractive index of the sample using a low-coherence white-light source [Fig. 1(d)].^13^^–^^15^ This backscattered signal interferes with a reference signal and can be used to extract quantitative phase information along the axial optical path length profile of the sample. It has been shown both theoretically and computationally that such a depth-resolved phase is most sensitive to the local variation of refractive index from its average value and the spatial heterogeneity in these variations, which are captured through the spatial frequencies inherent in the refractive index distribution of the sample.^13^^–^^15^ An example of transmission QPI and reflection QPI from weakly varying refractive indices within samples is shown in Fig. 3. While the image from the transmission QPI exhibits a high contrast [Fig. 3(a)], the image from the reflection QPI shows local changes of refractive index variation [Figs. 3(c) and 3(d)] in the depth-resolved manner.

### 2.2 Phase Retrieval

Phase retrieval techniques in QPI can be generally divided into two main categories: interferometric and non-interferometric methods.

#### 2.2.1 Interferometric methods

Temporal and spatial phase modulations are the most common among the interferometric methods. Temporal phase modulation, such as the phase shifting method, involves capturing a series of images with phase shifts at an increment of $\frac{\pi }{2}$ for each image for a four-step phase-shifting method. The sample-induced phase difference φ(x,y), is calculated using the following equation:

$$\varphi (x,y)=a\;\tan\;2(I(x,y,0)-I(x,y,\pi ),I(x,y,\frac{3\pi }{2})-I(x,y,\frac{\pi }{2})),$$

where a tan2 represents the arctangent function that calculates phase angles across a full circle range of [−π,π]. Alternative phase-shifting methods using a smaller number of phase-shifting steps^16^^,^^17^ are also possible for optimal balance between the accuracy of phase retrieval and imaging speed.

Spatial phase modulation is exemplified by off-axis holographic microscopy, often based on the Mach-Zehnder interferometer. This approach superposes a sample wave and a reference wave positioned at an off-axis angle θ, creating spatial interference fringes. The recorded intensity at the detector can be expressed as

$$I(x,y)=|E_{r}(x,y){|}^{2}+|E_{s}(x,y){|}^{2}+2|E_{r}(x,y)||E_{s}(x,y)|\cos[\varphi (x,y)+k_{x}x],$$

where $k_{x}$ is the spatial modulation frequency along the x axis, for example. This expression includes the zeroth order (DC component) (the first two terms) and the first diffraction orders. The off-axis configuration facilitates spatial separation of different diffraction orders in the Fourier plane, where one of the diffraction orders can be selected via spatial filtering. The filtered Fourier spectrum is then shifted to center at zero Fourier frequency to remove the modulation frequency $k_{x}$, and the phase induced by the object φ(x,y) is then extracted using a simple inverse tangent operation.

These two interferometric approaches have their individual advantages and limitations. Temporal modulation method has a better spatial resolution but at a compromised temporal resolution. While spatial modulation method allows a single snapshot image acquisition but sacrificing spatial resolution due to the need for fringes with higher spatial noise.

#### 2.2.2 Non-interferometric methods

Non-interferometric phase retrieval^18^ is a computational imaging approach, increasingly popular in recent QPI applications. In contrast to interferometric techniques, non-interferometric methods extract phase information directly from intensity measurements of transmitted light across multiple axial planes, eliminating the need for separate reference waves. Typically, a nearly collimated (partially coherent) illumination beam is used, to create an implicit interference between the scattered waves from the sample and the background. Among different approaches for phase retrieval directly from intensity measurements, the transport of intensity equation (TIE) is one of the most used methods. For an in-depth tutorial on TIE’s principles and applications for QPI, readers are directed to existing literature.^19^

The use of nearly coherent illumination inherently in non-interferometric phase retrieval methods limits the spatial frequency range covered, thus reducing lateral spatial resolution. However, this limitation can be mitigated through a synthetic aperture approach. Integrating waves from varying illumination angles, combined with direct or iterative algorithms for image reconstruction, it is possible to achieve an enhanced image resolution in the lateral direction. Importantly, this approach achieves a high lateral resolution while utilizing a low NA objective, thereby also expanding the field of view.^20^ Such a synthetic aperture approach is especially beneficial in applications where both high resolution and a large field of view are critical such as the detection of rare events in a large cell population.^21^

A major advantage of non-interferometric methods is their simple experimental setup, in a similar form of bright-field microscope without introducing additional optical components. In addition, unlike interferometric phase retrieval, these methods do not require phase unwrapping. However, the TIE-based computational phase retrieval often suffers from cloud-like low-frequency spatial noise, limiting its quantitative accuracy for recovered phase. As a result, the interferometry-based QPI remains the most accurate approach compared to the non-interferometric computational phase retrieval.^19^

### 2.3 Three-dimensional QPI

Traditionally, transmission QPI has predominantly focused on imaging 2D thin samples, where the phase is from the focal plane, or the cumulative optical path length is obtained as light travels through the sample. While 2D imaging provides valuable information on the cellular structure and physical parameters, it often misses the rich details of the 3D internal structures of the cells. With the emergence of 3D cell cultures and tissue models that more accurately recapitulate *in-vivo* conditions of human diseases, there has been a significant increase in the demand for methods that can visualize and analyze samples in their full 3D context while preserving their functional integrity.

Optical sectioning is a crucial aspect of 3D microscopy. However, traditional phase microscopy, typically configured in a wide-field transmission mode, is limited by the spatial frequency support of its optical transfer function (Fig. 2), resulting in poor axial resolution and suboptimal optical sectioning capabilities.^22^ Despite this limitation, certain phase microscopy techniques do offer some degree of depth sectioning, thanks to their use of phase-gradient–based image contrast. Techniques such as differential interference contrast (DIC) and various forms of oblique field microscopy (e.g., Hoffman modulation contrast,^23^ differential phase contrast^24^) are some notable examples. As the phase gradient is the highest at the focal plane, it provides some degree of depth sectioning capability for 3D imaging of phase objects.

Using a high-NA objective, ideally of the immersion type, is crucial for achieving the high axial resolution necessary for effective optical sectioning in 3D phase imaging. A simple and straightforward way to realize 3D phase imaging is to take a series of 2D images by sequentially moving the sample or the objective along the axial direction. The methods for retrieving quantitative phase information at each focal plane are like that used in 2D imaging. For example, phase-shifting interferometry,^25^ when applied in conjunction with conventional phase microscopies, such as Zernike phase contrast microscopy^26^ and differential interference microscopy,^27^ and paired with a high-NA objective, allows for reconstruction of high-resolution 3D phase images.

The most popular technical implementation of 3D phase imaging is optical diffraction tomography (ODT). The basic theoretical framework for reconstructing of 3D structure of a weakly scattering semi-transparent object via holograms was formulated by Emil Wolf in 1969.^28^ The 3D refractive index of the object can be retrieved from the complex amplitude distribution of the scattered fields, determined by measuring the intensity transmission functions of holograms at multiple illumination angles. Under the first-order Born approximation, applicable for weakly scattering objects, the 3D scattering potential of an object can be reconstructed from the collection of all Fourier components (or spatial frequencies) of the scattering potential in the K-space.^29^ The accuracy of this reconstruction depends on the accessible range in the 3D Fourier space, which can be obtained mostly by varying direction of incidence and/or wavelength. Most ODT setups, using a transmission configuration at a fixed wavelength, vary angles of illumination via rotation of samples or of illumination beam. By capturing the complex amplitude for all accessible spatial frequencies, the 3D scattering potential or refractive index can be reconstructed using methods like digital holography^30^^–^^34^ or computational phase retrieval.^35^ The resolution of the resulting image depends on the extent of coverage in the 3D spatial frequency space, which can potentially be limited by the “missing cone” problem [Fig. 2(b)]. There are limits to how much one can vary the angle of illumination due to physical constraints, such as the NA of the objective lens and potential blocking effects by the sample holder or the surrounding medium. Nevertheless, a high-resolution reconstruction of the 3D scattering potential of a biological cell at approximately 90 nm resolution, has been demonstrated by using two opposing high-NA (NA~1.4) objectives and a large coverage of spatial frequencies.^36^

Besides the advantage of superior resolution and 3D mapping of the scattering object, another important capability of ODT is its ability to measure the 3D refractive index (RI) distribution within cells. This feature distinguishes ODT from 2D QPI, where the measured phase shift is a product of the refractive index contrast and the physical length through the specimen. Thus, the phase shift inherently mixes information about the thickness and its refractive index of the sample.

### 2.4 Phase Noise

Phase is inherently highly sensitive to minute changes in optical path length, even down to the nanometer scale. This sensitivity, while enabling the detection of minute displacements in nanoscale structures, also makes phase measurements highly prone to various sources of noise. Some interferometry-based QPI systems require a separate reference and sample path, which is prone to vibration and temporal noise that hamper our ability to measure nanoscale dynamic changes of the cells.^5^ Moreover, the use of coherent laser introduces high speckle noise into the phase image which can degrade image resolution.

To achieve the desired nanometer or sub-nanometer sensitivity in phase imaging systems, it is crucial to minimize these sources of noise. A highly stable environment free from vibrations and thermal fluctuations is highly desirable. In addition, the use of actively stabilizing feedback loops is often necessary to minimize phase drift during image acquisition.^5^ By continuously monitoring the phase and making real-time adjustments to the optical path lengths, stable and accurate phase measurements can be achieved.

A significant advancement in the field of phase imaging to address the challenge of phase noise came with the development of the near common-path configuration such as in Fourier phase microscopy^37^^,^^38^ and diffraction phase microscopy.^39^ This setup, by making the reference and sample waves share nearly the same path, inherently reduces the sensitivity to variations in optical path length caused by external factors, such as vibrations and thermal fluctuations, effectively minimizing phase noise. This simple yet powerful approach facilitates its implementation in regular laboratory environments, making the near common-path configuration a popular choice for QPI systems, especially when high sensitivity is required for measuring nanoscale changes in cellular dynamics.

The introduction of low-coherence light sources, such as light-emitting diodes (LEDs) or lamps, has further contributed to a reduction in speckle and spatial phase noise^25^ and improved spatial resolution in imaging cells and tissue. Examples include use of a lamp in spatial light interference microscopy^25^ and quadriwave lateral shearing interferometry,^40^ use of low-coherence laser illumination in DHM,^41^ and use of LED in off-axis digital holography.^42^ In addition, a rotating diffuser^43^^,^^44^ coupled with the laser source has also been shown to reduce the spatial phase noise, while providing high-contrast interference fringes in an off-axis holographic interferometry system.

Alongside the strategies outlined previously to reduce phase noise, two additional crucial factors play crucial roles in further enhancing phase sensitivity. The first is maximizing the number of photons collected by the detector. This aspect is directly related to the characteristics of photon shot noise, which is governed by Poisson statistics and scales with the square root of the number of detected photons $\sqrt{N}$. By employing a strong light interference signal and pairing it with a camera that boasts a large full well capacity,^45^ it is possible to maximize the number of photons collected. This, in turn, serves to minimize the phase noise. The second critical factor involves the process of averaging multiple phase images. Remarkably, even averaging less than 10 frames can lead to a dramatic reduction in phase noise, often by a factor of around 2 to 3.^45^ Phase averaging is especially beneficial in applications that demand high phase sensitivity, such as measurement of the dynamics of neural deformation during the action potential.^46^ This approach is routinely used across various applications to achieve superior precision in phase measurements.

## 3 Applications of QPI for Precision Cancer Medicine

QPI is increasingly being recognized as a powerful quantitative imaging technology for basic biomedical research and clinical applications. While fluorescence microscopy continues to be a predominant microscopy technique in the biomedical community, QPI distinguishes itself with two primary attributes: its capability for label-free and non-invasive imaging of cells over extended periods and its exceptional nanoscale sensitivity to detect minute cellular and sub-cellular structure and dynamics.

In contrast to fluorescence imaging techniques that often requires fluorophores for visualizing specific targets, QPI offers intrinsic morphological contrast derived from the refractive index profile and physical thickness of weakly scattered cells and tissues. This label-free approach reveals detailed aspects of cell size, shape, volume, and refractive index in their native states. Importantly, QPI overcomes the issue of photobleaching, a common limitation in fluorescence imaging, enabling continuous observation of cells and tissues without the interference or potential toxicity of external labels or dyes. This feature is especially valuable for long-term studies, as it allows researchers to monitor cellular processes and morphological changes over a long period of time, from days to weeks.

Furthermore, the ability of QPI to detect changes in optical path length and refractive index at the nanoscale, even below the diffraction limit of the optical imaging system, is a simple label-free alternative to detect structural changes below the resolution limit. This nanoscale sensitivity facilitates the measurement of subtle changes in cells and tissues for a long time with millisecond temporal resolution, such as monitoring minute cell membrane fluctuations,^39^ tracking biomass changes during cell growth^47^ and observing neural signaling,^46^ all achievable without the need for labeling or using super-resolution imaging techniques. Additionally, its ability to detect subtle nanoscale structural changes on clinically prepared tissue section has shown promise in applications for cancer risk stratification^14^^,^^48^^,^^49^ and prognosis.^50^^,^^51^

The wide-ranging applications of QPI extend from fundamental cell and developmental biology to neuroscience and clinical diagnosis. The extensive implications and diverse applications of QPI in biomedicine have been thoroughly reviewed in recent literature.^52^^,^^53^ This section, however, will specifically focus on the potential role of QPI in enhancing cancer detection, prevention, and treatment.

### 3.1 Precision Oncology

Precision oncology represents a paradigm shift in cancer treatment, moving away from one-size-fits-all approaches to tailored treatments based on individual patient’s characteristics. Central to this approach is the goal of identifying the most effective treatment for each patient. This process necessitates a comprehensive understanding of unique characteristics (e.g., molecular profiles and biophysical properties) of a patient’s tumor, as well as its responsiveness to a diverse array of therapeutic agents. The emergence of patient-derived primary cell cultures and organoids has opened new avenues for personalized treatment selection by enabling the testing of a broad spectrum of therapeutic agents directly on the patient’s own tumor cells. In this context, there is a growing need for non-invasive technologies capable of precisely assessing treatment responses.

Ideally, these technologies should provide precise and undisturbed measurements to assess how a patient’s tumor cells respond to various treatments without the need for additional dyes or markers that might otherwise influence the drug’s action. While fluorescence imaging has proven effective in such applications,^54^ the use of common fluorescent labels can present its own challenges. Notably, these labels can induce DNA damage, which is also a mechanism employed by many chemotherapy drugs, thereby complicating the distinction between the cytotoxic effects of the treatment and potential artifacts introduced by the labels.^55^ Although many fluorophores are designed to minimally perturb biological functions, considerable effort is often necessary to rule out their cytotoxic effects. QPI has the potential to address this challenge by providing a direct view of cellular responses to cancer therapies in their native state, which allows for a more accurate assessment of the actual effects of treatments on tumor cells. Concurrently, fluorescence imaging is highly effective for uncovering the underlying molecular mechanisms behind diverse treatment responses and for identifying novel targets for more effective therapies.

Cancer is characterized by uncontrolled cell proliferation, a primary target of many cancer therapies. QPI offers a non-invasive, label-free, and precise method to quantify cell mass, a key biophysical parameter for assessing cell growth and death. Real-time monitoring of cell mass dynamics in response to pharmacological agents over extended periods provides essential insights for the action of drugs and evaluating cancer treatments.^56^^–^^58^ Quantitative assessment of cell dry mass using phase microscopy can be traced back by the foundational work by Barer.^59^ The total dry mass (m) of a cell can be estimated using the measured phase of the cell as follows: $m=\frac{1}{\alpha }\underset{S}{\iint }\mathrm{OPL}(x,y)dx\;dy$. Here, S represents the area of the cell and α denotes a constant known as the specific refraction increment. For most biological cells, α typically lies between 0.18 to 0.21  mg/ml. Therefore, dry mass can be determined through the quantifiable phase and cell area. QPI also enables the measurement of other cellular and subcellular attributes like volume, surface area, thickness, and refractive index in a non-invasive manner.^60^^,^^61^

**Fig. 4:**
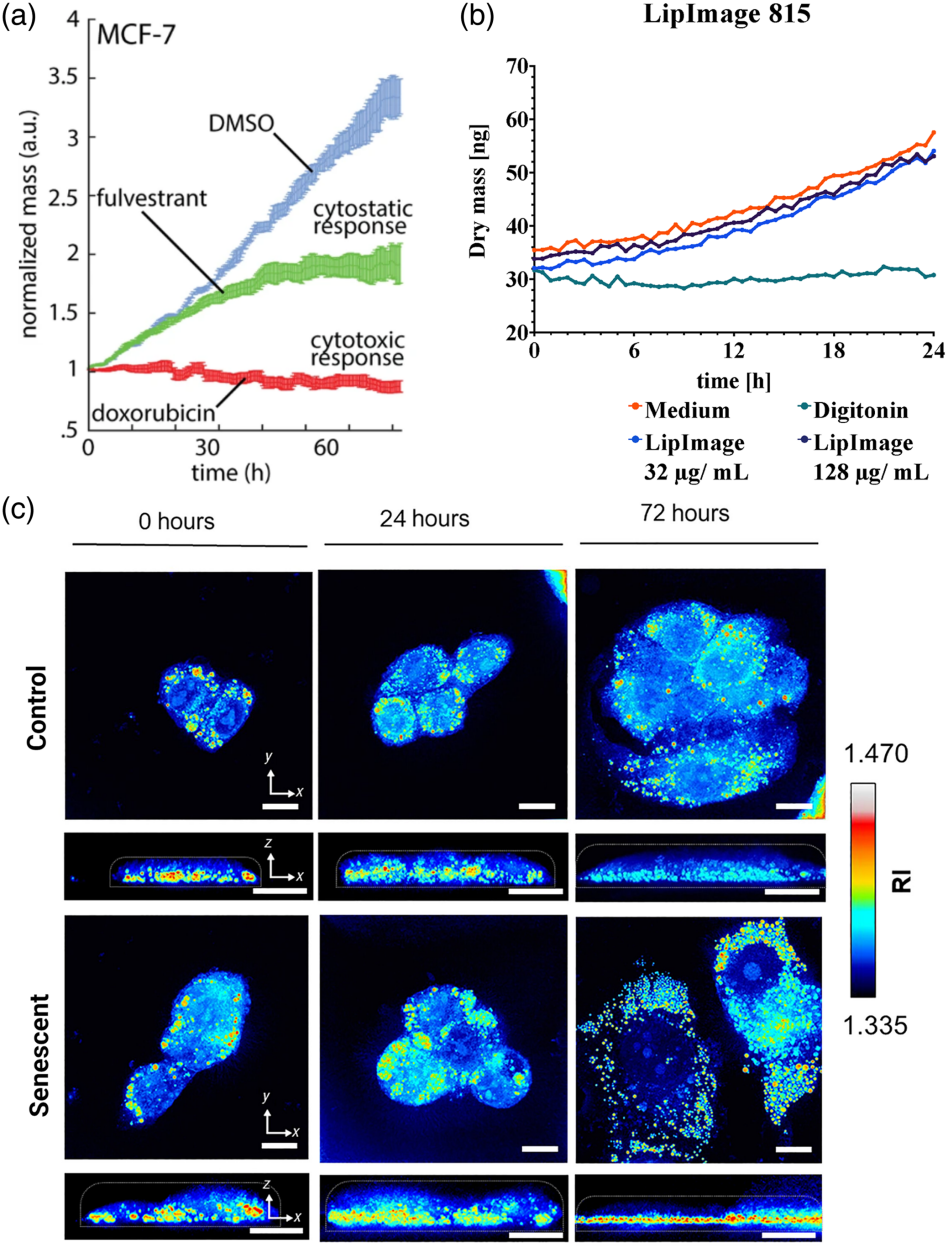
Examples for the applications of QPI in assessing drug response. (a) The fold change of average cell dry mass in MCF-7 cells over 72 h of treatment with 20  μM doxorubicin (cytotoxic response, slow decrease in dry mass) and 20  μM fulvestrant (cytostatic response, gradual increase in dry mass) in comparison with the control (DMSO), adapted with permission from Ref. 58. (b) Temporal changes of cell dry mass in macrophages (RAW 264.7) treated with different concentrations of lipid-based nanoparticles (LipImage 815), compared to cytotoxicity and medium controls. The results suggest LipImage 815 as low-toxic. The figure is adapted from Ref. 63. (c) The 3D refractive index maps in cells undergoing regular proliferation (control) and senescent cells. Senescent cells showed more lipid droplet accumulation compared to controls (those with the highest refractive index). The figure is adapted with permission from Ref. 61.

A key function of cytotoxic drugs in cancer treatment is to inhibit cell growth. Monitoring dynamic changes in cell dry mass through QPI provides a straightforward method to assess the cytotoxic responses elicited by various drugs and nanomaterials.^58^^,^^62^^,^^63^ Typically, an increase in cell mass before cell division is indicative of cell growth. However, when exposed to cytotoxic drugs at effective concentrations, a stagnation or even reduction in cell dry mass can often be observed over the treatment period, which can span several days^58^ [Fig. 4(a)]. Similar approaches with QPI can also be used to evaluate cell viability^63^ [Fig. 4(b)], distinguishing between live and dead cells.^64^

This capability of QPI is particularly valuable in the context of drug screening. It enables researchers to quantitatively determine the effects of new treatments on cancer cells. Frequently, QPI systems are integrated with multi-well plates, a standard format in drug screening, allowing simultaneous evaluation of various drugs at different concentrations in a single experimental setup. Continuous monitoring of cell mass through QPI provides immediate insights into cellular responses to diverse drug concentrations and combinations, helping to identify different drug mechanisms of action. For instance, cytostatic drugs, which disrupt cell signaling and replication without immediately reducing tumor burden,^65^ demonstrate a slower increase in cell mass, in contrast to the static or diminishing cell mass dynamics typically induced by cytotoxic drugs^58^ [Fig. 4(a)]. Moreover, 3D refractive index tomography combined with other label-free imaging techniques has been used to explore therapy-induced senescence, which holds immense potential for identifying the most effective anti-cancer treatments in the future.^61^

### 3.2 Precision Cancer Prevention, Diagnosis, and Prognosis

Alongside its applications in dynamic cellular imaging, QPI has emerged as a promising technique for cancer detection and prognosis. This is largely attributed to its ability to detect nanoscale structural changes in clinically prepared tissue sections or cytology samples. Historically, cancer diagnosis has relied on the microscopic examination of tissue architecture and nuclear features, typically visualized on hematoxylin and eosin (H&E)-stained slides through conventional bright-field microscopy. Despite its long-standing application, this method faces limitations due to its diffraction-limited resolution and dependence on absorption-based contrast. These limitations can result in missing subtle cellular abnormalities that emerge in the early stages of carcinogenesis or in cells with a high risk of progressing to cancer. In contrast, QPI provides detailed quantitative phase information related to cell mass or nanoscopic structural changes, thereby offering sensitive measures for detecting malignant transformation or progression, significantly enhancing early cancer detection and prognosis.

Transmission QPI produces high-contrast images of tissue architecture, resulting from the cumulative phase shift across a sample’s thickness. Regions with high density or refractive index, such as the extracellular matrix, often contribute to this high contrast. For example, QPI-derived metrics of light scattering anisotropy in stromal areas adjacent to cancerous prostate glands have been successful in stratifying patients by recurrence risk of prostate cancer.^50^ The use of quantitative image analysis, like fiber tracking algorithms, to evaluate the structural composition of the extracellular matrix in pancreatic adenocarcinoma, has demonstrated significant prognostic implications. Variance in tissue refractive index has also emerged as a promising marker for distinguishing between tumor and normal tissues.^66^ Importantly, the recent emergence of convolutional neural networks has further empowered the application of QPI in histopathology, enabling the classification of benign versus cancerous tissue with high accuracy directly from phase images.^67^ This AI-enhanced analysis of QPI provides a unique physical contrast and diagnostic information that complements traditional H&E staining, introducing an additional, powerful modality to digital pathology.^68^

**Fig. 5:**
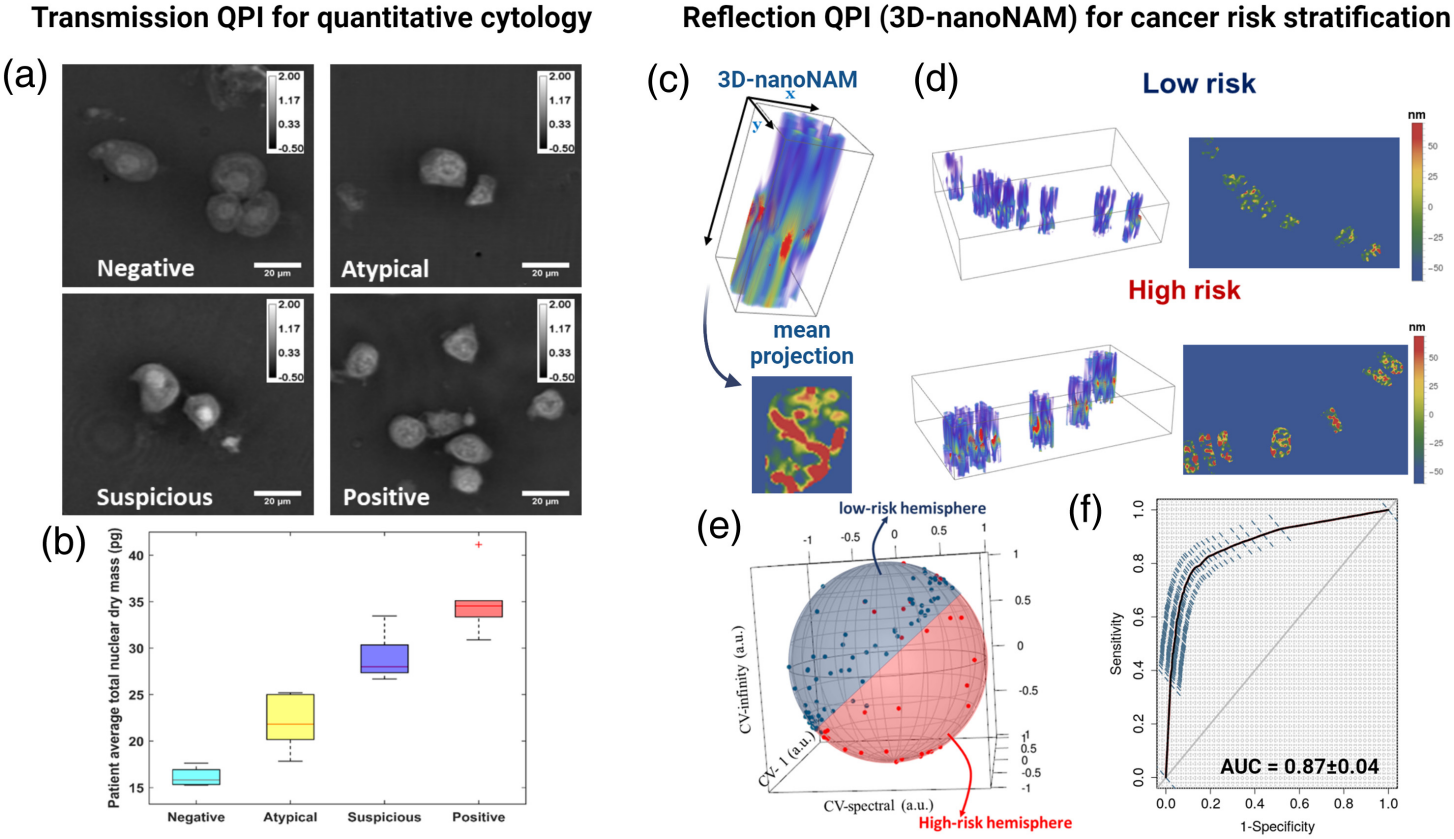
Examples for the applications of QPI for quantitative diagnosis and precision prevention. (a) Transmission quantitative phase images for urine cytology samples (negative, atypical, suspicious, and positive classified by an expert cytopathologist). (b) The average nuclear dry mass for each patient group. (c)–(f) Application of reflection QPI for assessing 3D nanoscale nuclear architecture for cancer risk stratification in patients with ulcerative colitis. The figures were adapted from Ref. 69. (c) and (d) 3D-nanoNAM from a low-risk and a high-risk patient. (e) The 3D-nanoNAM derived properties projected onto a unit 2-sphere, where most of the low- and high-risk patients lie on two separate hemispheres. (f) The area under receiving operating curve (ROC) for distinguishing the low- from high-risk patients is 0.87±0.04. The figures were adapted from Ref. 48.

At the cytology level, transmission QPI enables precise measurements of nuclear or cellular dry mass. The nuclear dry mass has shown strong correlation with urine cytological diagnoses, where increased average nuclear dry mass and increased entropy of dry mass in the nuclei were consistently observed in cancer cells^69^ [Figs. 5(a) and 5(b)]. These characteristics reflect the enlarged nuclei size and increased cell proliferation typical in cancer. The QPI-based assessment offers the potential to significantly reduce inter-observer variability and enhance diagnostic accuracy in pathology. Moreover, computational imaging-based QPI facilitates the reconstruction of quantitative phase images over a large field of view without the need for mechanical scanning.^70^^–^^72^ Such computational approaches are particularly advantageous as they enable in-focus imaging through numerical refocusing, a critical feature for cytology samples where cell clusters can complicate accurate focusing.^73^ The simplicity of computational imaging-based QPI also allows for its integration into mobile phones or chip-based devices,^70^^,^^74^ showing its potential as a low-cost, point-of-care solution for cytological diagnosis. This adaptability makes QPI a versatile tool, capable of bringing high-quality, diagnostic imaging capabilities to remote or resource-limited settings, thus broadening the accessibility and impact of advanced diagnostic technologies.

QPI-based imaging cytometry also emerges as a novel solution for point-of-care and high-throughput diagnostics.^70^^,^^73^ This technique integrates microfluidic chips with QPI, enabling ultrahigh-throughput, label-free analysis that generates detailed refractive index maps at the single-cell level even with some subcellular details, analyzing thousands to hundreds of thousands of cells per minute.^75^^–^^77^ This approach is particularly effective for analyzing non-adherent cells, such as those from bodily fluids or fine needle aspirations, eliminating the need for monolayer cell preparation on coverslips. For example, holographic cytometry has significantly advanced single-cell analysis by allowing for the detailed profiling of biophysical phenotypes or AI-assisted analysis across large populations to distinguish different cell types and pinpointing early-stage cancers^78^ and identify drug-resistant cancer cells.^79^

Another significant area of clinical application lies in the use of reflection QPI for detecting local refractive index variations, which serve as sensitive markers for cancer risk. Cells in the early stages of carcinogenesis, despite having an increased risk of developing cancer, often do not display microscopically discernible structural changes and are thus categorized as having the same pathological phenotype (e.g., normal-appearing or as similar types of precursors). However, these cells may exhibit distinct molecular characteristics, such as more disrupted chromatin compaction, indicative of a higher level of genomic instability that can drive carcinogenesis.^80^ The detection of such molecular-scale changes in nuclear architecture necessitates a technique capable of identifying local variations in refractive index, which could be sensitive indicators of changes in chromatin compaction.

Reflection QPI is well suited for this task, particularly in the weakly varying scattering medium without a strong interface. While it may not capture information about the average refractive index and might not offer as high an image contrast as transmission QPI, reflection QPI excels in detecting local variations in the refractive index profile of the sample as a function of its depth-resolved spatial frequencies.^15^ Its depth-resolved nature and sensitivity to nanoscale changes enable the examination of local structural heterogeneity, independent of sample thickness.^13^^,^^14^ When this method is combined with high-contrast image from transmission QPI and standard histology, the resulting approach—termed nanoscale nuclear architecture mapping (nanoNAM)—has demonstrated its ability to distinguish low-risk from high-risk patients with ulcerative colitis^14^^,^^48^ and Barrett’s esophagus,^49^ even from normal-appearing or non-neoplastic tissue, highlighting its potential as a tool for risk stratification and precision surveillance in a large patient population at increased risk for developing cancer.

Besides the static structural characteristics, measuring cellular dynamics as cancer biomarkers offers a novel and highly sensitive approach to understanding and assessing cancer cells. QPI captures the spatial and temporal fluctuation of optical path length and cell mechanical signatures such as shear modulus and stiffness, which were shown to distinguish cancer cells with different malignant potential.^81^^–^^84^ Cell deformation and migration, quantitatively assessed through QPI, enable the measurement of matrix stiffness^85^ and viscosity,^86^ offering critical insights into the dynamic behaviors of cancer cells. These measurements could assist grading of metastatic potential and development of patient-specific therapeutic strategies. Collectively, QPI assessment of pathological cells and tissue offers valuable insights in cancer prevention, diagnosis, and prognosis, leveraging its precise quantitative imaging capabilities to inform clinical decisions.

### 3.3 *In-vivo* Tissue Imaging

Transmission QPI has primarily been applied to cell imaging in samples that are only several microns thick, largely due to its transmission configuration which optimally captures phase information from thin specimens where the transmitted light can pass through with minimal multiple scattering. However, transmission-like phase information can be obtained using reflection geometry, which is highly advantageous in imaging thick tissue.

Oblique back-illumination microscopy,^87^ a form of oblique-field microscopy,^22^ operates on the principle that oblique illumination beam introduces asymmetry from the detection pupil, resulting in phase-gradient image contrast. This light propagates inside the sample, undergoes multiple scatterings by the tissue, and eventually returns to the detector. This process effectively transforms what initially appears as back illumination into trans-illumination. This elegant approach used in reflection geometry can provide information analogous to what one might obtain with transmission QPI and generate high-contrast images based on the cumulative optical path length, thus enabling effective phase imaging even in thick tissue samples. As discussed earlier in the context of 3D QPI, the highest contrast in the phase gradient is achieved at the focal plane, granting this method with a certain degree of optical sectioning capability. This approach has been explored to provide histology-like contrast on surgically resected tissue,^88^ which holds great potential for real-time tumor margin assessment. This adaptation of QPI for thicker tissue imaging underscores its versatility and applicability in a range of clinical settings, particularly in surgical oncology.

## 4 Challenges and new opportunities

### 4.1 Molecular specificity

QPI’s unparalleled ability to capture detailed cellular morphology, at nanoscale sensitivity below the diffraction-limited resolution, in a non-invasive and label-free manner opens up possibilities for observing phenomena that might not be possible using other techniques. But the lack of molecular specificity is the most notable limitation of QPI and poses a substantial challenge in correlating phase image features with its underlying molecular composition of the cells, specific biological structures, processes, and pathologies—a task that is more directly accomplished by fluorescence microscopy with its targeted molecular imaging capabilities.

The quest for molecular specificity in QPI imaging has spurred researchers to seek out novel methods that could bridge this gap. The advent of artificial intelligence (AI) offers a promising solution to this challenge. By leveraging advanced computational algorithms and machine learning methods, it is possible to infer molecular information from the complex phase images. By using training datasets that match fluorescence with quantitative phase images, AI models can establish connections between QPI signatures and molecular markers. This approach has led to the identification of specific structural attributes in QPI images that correspond to their fluorescent counterparts, effectively providing QPI with an element of molecular specificity.^89^^–^^91^ This enhancement could significantly broaden the utility of QPI and increase its applicability in molecular-focused biomedical research. Additionally, AI can convert label-free QPI images into traditional H&E stained histology-like images,^92^^,^^93^ making the interpretation more intuitive for pathologists.

However, the accuracy of predicting molecular structures from QPI images often depends on their level of contrast. Features such as cell nuclei and lipid droplets, which are prominent in QPI, benefit from this AI-enhanced virtual staining. This process introduces a level of molecular detail to QPI’s morphological analysis. In contrast, molecular structures with variable contrast due to cellular functional states may not be as precisely predicted. Therefore, while promising, this technique must be applied with caution, especially when dealing with novel biological processes not included in the training dataset or highly heterogeneous cellular states. Such cautious application ensures that the AI-assisted phase staining technique remains a reliable augmentation of QPI’s capabilities within its established biological contexts.

An emerging direction in the QPI field is moving beyond the simple correlation of specific molecular structures with QPI-derived morphological features or converting QPI data into analogous fluorescence images. The true strength of QPI lies in its unbiased presentation of cellular and subcellular morphology, not limited by labeled targets. This can then be linked to the functional states of individual cells. Noteworthy examples are the accurate classification of cell cycle phases,^94^ the identification of apoptotic cells,^95^ and the detection of cell death.^64^ These advancements underscore the QPI’s potential to reveal underlying biological processes in a non-invasive and label-free manner.

### 4.2 Bridging Dynamic QPI Imaging of Cell Morphology with Precision Cancer Medicine

Cell morphology, the study of cell shape, size, and structure, plays an important role in all stages of cancer development. Morphological changes in cells are increasingly recognized as a direct, measurable reflection of the intricate molecular underpinnings of cancer, serving as a window into the cellular behaviors and transformations that underlie malignancy.^96^ The dynamic nature of cell morphology, therefore, becomes a powerful tool in both understanding and combating cancer.

Building on the foundational importance of cell morphology in cancer development, dynamic QPI is set to transform morpho-dynamic studies, leveraging its nanoscale precision and quantitative capabilities. QPI’s ability to provide an unbiased view of cellular and subcellular morphology at nanoscale sensitivity, when observed over extended periods, captures the complex narratives of cellular evolution. This includes tracking growth, division, differentiation, malignant transformation and in response to different environmental stimuli, all while preserving the natural functions of cells. Future advancements in imaging throughput, resolution, and 3D capabilities will further enable the observation of dynamic cellular events with high temporal and spatial resolution across various spatial scales over a large cell population, allowing for the monitoring of a vast array of cellular processes.

The next significant step is to correlate the rich spatial and temporal data derived from QPI with the broader context of cellular behavior and ultimate cell fates. Analyzing dynamic morphological characteristics across tens of thousands of cells presents a complex challenge, one that traditional quantitative analysis using a limited set of morphological metrics may struggle to address. This is where the emergence of artificial intelligence (AI) becomes instrumental. The integration of QPI with computational models and machine learning is key in uncovering cellular characteristics that predict complex behaviors and phenotypes. For example, melanoma cells with different metastatic potential exhibit distinct dynamic behaviors of morphological characteristics, which are too subtle for human observation but can be accurately identified by AI algorithms.^97^

**Fig. 6:**
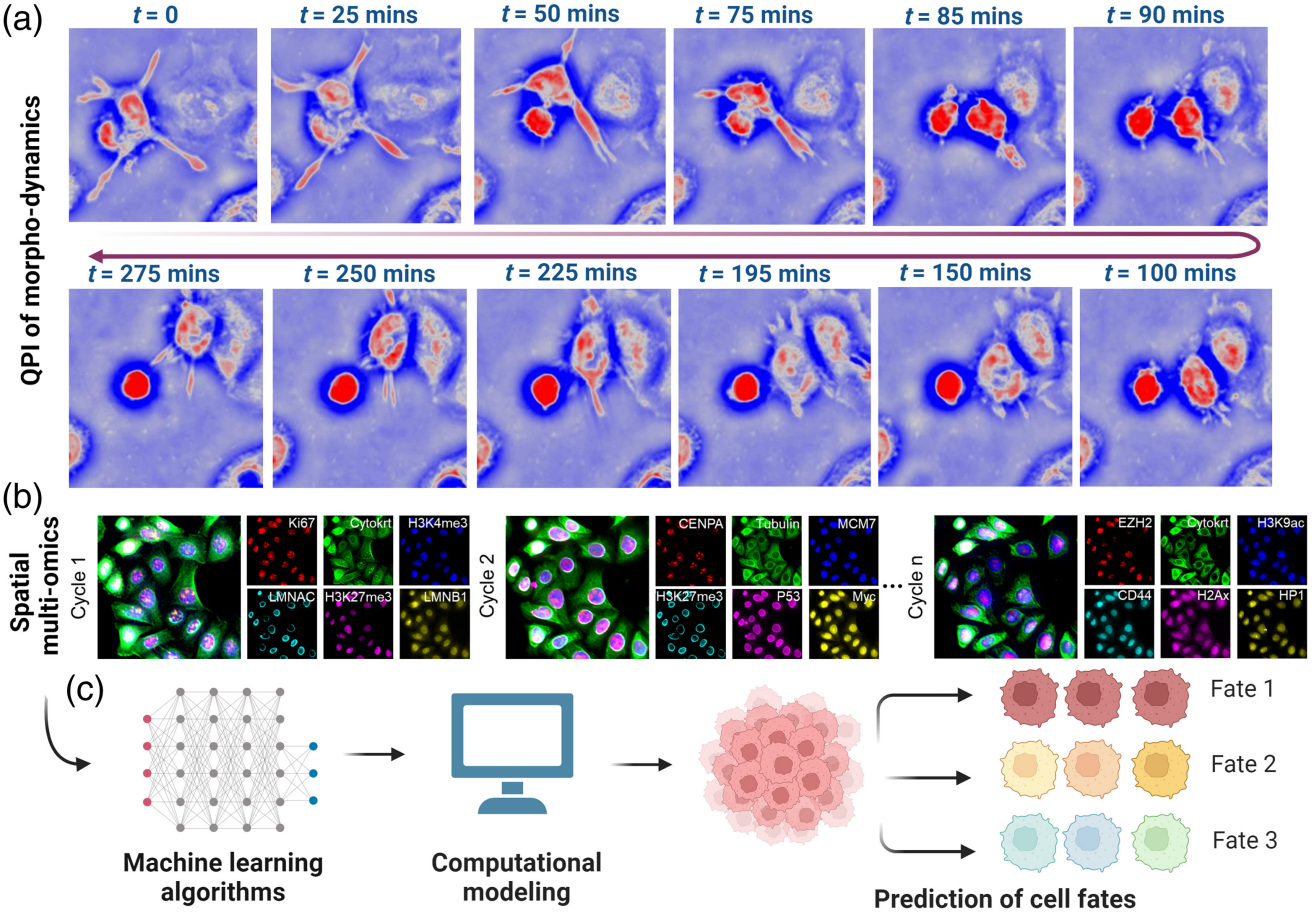
Conceptual framework for bridging dynamic QPI imaging of cell morphology to improve precision cancer medicine. (a)  Dynamic morphological changes of cellular and subcellular structures over an extended period, as shown in this representative image sequence which illustrates the plastic nature of cell morphology in cells, coupled with (b) spatial multi-omics performed on the same cell population, and (c) assisted with AI and computational modeling, have the potential to predict cell fate decisions.

However, the information about dynamic cell morphology changes over time, while valuable, is not sufficient on its own. It is critical to rigorously connect morphological trajectories with underlying molecular characteristics and phenotypic outcomes. This foundational step requires integrating morphological data with corresponding spatial multi-omics and high-content phenotyping for individual cells of the same cell population within their spatial context. Combined with the analytical prowess of AI and systems biology, this integration offers powerful capability to predict cell fate decision at various cancer stages and in response to therapeutic agents.^98^ The conceptual framework is illustrated in Fig. 6. Once established, high-throughput morpho-dynamic imaging of patient-derived cell models could find many clinical applications. For example, it can used to identify early cellular alterations indicative of cancer progression that precedes the manifestation of traditional biomarkers or guide personalized medicine by matching therapeutic screening with precise cellular responses to tumors. Thus, integrating dynamic QPI imaging with the principles of precision cancer medicine could dramatically reshape our approach to diagnosing, monitoring, and treating cancer.^99^

### 4.3 Patient-derived Model Systems

Patient-relevant *in-vitro* tumor models have become increasingly essential in precision cancer medicine, evolving significantly from immortalized cancer cell lines to sophisticated organoids. Traditionally, cancer research relied on immortalized lines, which have been invaluable for studying cancer biology, drug screening, and understanding molecular mechanisms due to their ease of use and high reproducibility. However, their lack of genetic diversity and cellular heterogeneity often limits the translatability of research findings to clinical applications.

Primary cancer cell lines, derived directly from patient tumors, retain more characteristics of the original tumor, thereby enhancing the relevance of *in-vitro* models for personalized medicine. Moreover, the development of 3D models like tumor spheroids and organoids marked a significant leap. Tumor spheroids, as 3D cell cultures, replicate the structural, microenvironmental, and cell interaction complexities of tumors in the body, offering deeper insights into tumor growth, invasion, and drug resistance mechanisms. In particular, patient-derived organoids, miniaturized versions of organs grown *in vitro* directly from patient tumor samples, stand out as the most realistic *in-vitro* tumor models.^100^^–^^103^ They closely resemble the tissue architecture at various stages of tumorigenesis, cellular environment, and genetic makeup, making them invaluable for studying tumor biology,^104^ drug screening,^105^ and understanding patient-specific responses to therapies.^106^^–^^108^

QPI has established its utility in assessing the physical properties of 2D cell lines, such as in studying mass dynamics in response to drug treatments, as previously discussed. While its application in imaging 3D spheroids and organoids is still emerging, several promising studies have begun to showcase its potential. For instance, QPI-based mass dynamics, albeit at a single organoid resolution, have been recently used in bio-printed 3D organoid models of cancer to evaluate drug efficacy.^109^ Techniques like gradient light interference microscopy^27^ and its deep-learning assisted artificial confocal microscopy^110^ have demonstrated significant promise in imaging 3D spheroids. Additionally, ODT has been effective for long-term high-resolution imaging of 3D intestinal organoids.^111^

The development of high-throughput QPI systems that can achieve long-term, in-depth imaging of 3D organoids with sub-cellular resolution and contrast is a critical need in the field. The semi-transparent, thick 3D organoids introduce technical challenges, as multiple scattering and refractive index heterogeneity can cause aberrations and degrade image resolution. This complicates the acquisition of high-resolution 3D refractive index maps. To address these challenges, innovative theoretical models, computational image reconstruction algorithms, and experimental techniques are being developed.^35^^,^^112^^–^^115^ These strategies aim to mitigate the effects of multiple scattering and enhance imaging depth, such as using iterative back propagation and multi-slice models from an initial estimate of partial refractive index map to refine the reconstructed 3D refractive index maps^35^^,^^112^ or solving the inverse scattering problem using the more accurate theoretical models.^115^ The integration of physics-based deep learning methodologies with these techniques is proving to be particularly effective, offering enhanced accuracy in the reconstructed images.

Despite these advances, the image resolution and imaging depth remain to be improved to make QPI truly suitable for imaging 3D organoids and other complex scattering samples. The computational speed for reconstructing a large 3D scattering object is rather slow. Robust and accurate cell segmentation methods and software packages for 3D quantitative phase images, especially for cell aggregates, are still lacking.

The future integration of dynamic QPI with 3D imaging capabilities in these advanced *in-vitro* tumor models opens significant possibilities for label-free drug screening. By enabling non-perturbed analysis, this approach facilitates the identification and selection of the most effective therapies. The application of dynamic and 3D QPI in innovative tumor models represents a promising frontier in precision cancer medicine, offering the potential to revolutionize the way we evaluate and select cancer treatments.

### 4.4 Clinical Pathology

Conventional pathology has long been the cornerstone of cancer diagnostics, informing the important aspects of disease identification, staging, and prognosis for over two centuries. However, the landscape of pathology is undergoing a significant transformation with the advent of FDA-approved digital pathology platforms that, when fused with the analytical power of AI, are redefining the paradigms of pathological assessment. Adding to this, the emergence of spatial biology has become critical to unraveling molecular characteristics in the spatial context of tissue architecture and microenvironment to guide precision cancer medicine.

Within this evolving framework, QPI offers the potential to refine the diagnostic precision of traditional histopathology, particularly in areas where conventional techniques show limitations. The integration of QPI into clinical practice is seen as promising along three avenues: it bolsters the accuracy of cytological assessments, provides prognostic insights by profiling tissue microenvironments, and delivers quantitative analysis that enriches the morphological observations of standard histopathology, aiding in risk assessment of precursors.

QPI aligns well with current clinical workflows, utilizing sample preparations compatible with those of traditional histopathology or FFPE tissue blocks obtained as the standard of care. It introduces no disruption, such as the need for additional tissue collection. Transmission QPI offers enhanced contrast in visualizing tissue architectures, such as the extracellular matrix, that may be less prominent in conventional histological images. The biggest strength of QPI lies in its sensitivity to nanoscale structural alterations within cells and tissues, detecting changes that may precede those detectable by conventional methods. For example, the reflection QPI’s ability to identify aberrant nuclear architecture in early carcinogenesis as a valuable indicator for stratifying patients who may be at highest risk for cancer development.^13^^,^^14^^,^^48^^,^^49^

Beyond enhancing conventional 2D histopathology, QPI’s potential for 3D volumetric imaging addresses a critical need in histopathology for comprehensive 3D tissue evaluation. Traditional methods, while informative, often overlook the complex 3D microenvironment due to their reliance on thin tissue sections. While innovative technologies like serial section stitching (CODA)^116^ and light-sheet microscopy of cleared tissue^117^ have made significant progress towards achieving visualization of large-scale 3D tissue architecture with microscopic details, they are time-consuming and labor-intensive. Recent advances in label-free 3D volumetric mapping through ODT demonstrate QPI’s capability to produce detailed 3D refractive index maps of tissue.^118^ This innovation presents a new opportunity for QPI toward realizing efficient label-free 3D histopathological diagnosis.

Moving forward, the combination of spatial biology with QPI’s precise mapping of tissue architecture and nanoscale details could serve as a new and powerful approach. This integrative method promises a multi-scale, quantitative characterization of tumors and their precursors, extending from the molecular to the systemic level, potentially transforming our approach to precision cancer medicine. The future synergy between AI and QPI also enriches the diagnostic process and paves the way for advancing multi-modal digital pathology to a new level of precision and insight.

Nevertheless, the path to its widespread clinical adoption is filled with challenges. A paramount challenge is the standardization of QPI protocols. Different types of QPI methodologies and image reconstruction algorithms can yield variable results. Hence, for a universally accepted set of procedures and algorithms that guarantee reproducible outcomes, irrespective of the laboratory or equipment utilized. Furthermore, clinical samples, inherently highly processed, necessitate QPI methodologies equipped with robust self-calibration and reference standards to accurately process and interpret the vast array of sample conditions encountered in a clinical setting. While QPI holds great promise, its integration into clinical practice will require a concerted effort to overcome technical and logistic challenges. Addressing these will ensure that QPI can become a vital element of contemporary medical practice, offering clinicians a more profound assessment of pathogenesis, and aiding in the delivery of targeted interventions.

## 5 Conclusion

In summary, QPI is emerging as a promising tool to improve precision cancer medicine. Offering label-free and nanoscale precision, QPI reveals detailed cellular and tissue structures in 2D and 3D, thereby enabling high-throughput, non-invasive and real-time monitoring of cellular dynamics. This opens new doors for understanding cancer behavior and its response to therapies. The dynamic imaging capabilities of QPI offer a live, real-time view into cellular processes to therapeutic agents, crucial for understanding the complex behaviors of cancer cells and the immediate effects of therapeutic agents. When integrated into drug screening, QPI’s insights into drug actions and responses offer a comprehensive understanding of treatment efficacy. Additionally, the rise of spatial biology and multi-omics provides a multidimensional view of the tumor microenvironment, and QPI has shown the potential to enhance these approaches by adding a layer of label-free spatial and morphological context to genomic, proteomic, and metabolomic data, leading to a more integrated understanding of cancer biology.

Despite its potential, there are numerous technical challenges to overcome for QPI to fully impact cancer research. Improvement in imaging throughput, resolution, and imaging depth, especially for 3D patient-derived tumor models, more accurate theoretical models for solving inverse scattering problems, accelerated image reconstruction algorithms, robust cell segmentation tools and seamless integration with other imaging techniques are imperative. Additionally, the development of accessible and user-friendly software for comprehensive image analysis and the standardization of QPI data analysis will pave the way for its broader application in the biomedical field. Finally, a proper understanding of label-free contrast images and what they represent in the heterogeneous context of cancer is currently lacking. Integration of QPI with other spatial omics approaches will go a long way in overcoming this barrier.

The future of QPI in advancing precision cancer medicine is bright. It holds the promise to deepen our knowledge of tumor biology, improve diagnostic and prognostic precision, help tailor treatments to individual patient profiles, and ultimately improve patient outcomes. As we stand at this technological forefront, the continued development and integration of QPI with cutting-edge biomedical research and clinical applications hold tremendous potential to transform cancer care.

## Data Availability

Data sharing is not applicable to this article, as no new data were analyzed.
